# Can We Transplant Conceptual Frameworks of Healthcare Quality Evaluation from Developed Countries into Developing Countries?

**DOI:** 10.4103/0970-0218.51223

**Published:** 2009-04

**Authors:** Sudha Ramani

**Affiliations:** Department of Health Policy and Management, Boston University School of Public Health, Boston

Quality assessments are slowly being recognized as integral components of new and existing healthcare programs in India. The adoption of the Indian Public Health Standards (IPHS) by the National Rural Health Mission is a hallmark initiative in this area. This paper provides a perspective on whether we can adapt sophisticated quality improvement techniques offered by developed countries to measure healthcare quality in our local contexts.

Classical models of quality evaluation measure quality in terms of three attributes: structure, process, and outcome.(1,2) Even while viewing quality as a multidimensional entity as conceptualized in the model, developing and developed countries might need to focus on different dimensions of quality. In developing countries, there is an inherent importance given to structural components of quality, stemming from a long history of structural inadequacies. For example, the IPHS largely addresses the structural lacunae such as the availability of medicines, equipment, etc. and there are few components that measure processes and none that measure outcomes. In resource-rich settings, quality measurements are becoming increasingly process and outcome oriented. But we cannot blindly employ the same indicators of quality measurement since improving processes and outcomes may prove impractical for us before basic structural competence is achieved.

However, even while accepting the importance of structural measurements, we need to understand the limitations of using structural measures as a proxy for measuring quality. A study by Ehiri, *et al.* assessed child health services in Nigeria using both structural and process measures. The study found that structural failings within the system were accompanied by important process failings - like not using the national case management algorithm and the lack of protocol for supervision of health workers.(3) This shows us that quality in developing countries is not completely structure dependent. If we use only structural measures of quality, there is a danger of *blaming the lack of quality entirely on the lack of structure* in spite of the existence of several deficiencies in the quality of care that are unrelated to structure. Though it is reasonable to have structure-oriented standards like the IPHS, there might be utility in including a few process and outcome measures among the structural measures. For example, a broad indicator on drug availability in the IPHS (structure-oriented) can be supplemented by specific measures on how a particular class of drugs is administered (process-oriented) and what benefits it fetches (outcome-oriented). The idea is to ensure that we do not ignore failed, non compliant processes that affect quality and also make certain that our structural improvement efforts are having some outcome effects.

The challenge, in our context, would be in choosing and standardizing relevant measures. Particularly while assessing processes, it is important to keep in mind that process variations from recommended standards (often set in developed countries) may have arisen in practice due to specific valid reasons. Processes are often modified to suit the healthcare environment automatically. It is important to deliberate on what makes processes operate differently in developing countries and importantly, if the existing variation in a given process is actually detrimental to quality.

Much about quality can be learned from different countries and settings. Adapting generic strategies and tools to provide local solutions for local problems can be considered an optimal way of solving quality problems in resource-constrained settings. The key point to remember while borrowing quality concepts is that we must use these as good starting points. It is imperative that we do not waste costly resources on re-inventing the quality wheel. At the same time, we must shape and size the wheel to suit our healthcare vehicle.

The most common misconception about “quality healthcare in developing countries is that it doesn't exist at all. It does exist, although probably it is restricted to certain locations. Samandari, *et al.* studied a privately owned eye-hospital in India that provides excellent quality care,(4) and describe how this hospital found practical ways of integrating quality monitoring with limited funding. Such innovations can be replicated and fit into similar settings. When resources are limited, it becomes essential to prioritize which concepts of quality would yield the best benefits. We need to carefully weigh if quality improvement techniques, especially the costlier ones (accreditation, certification) are worth the time and effort spent on them.

Lastly, a fundamental change in the perception of quality care needs to be brought about before quality measures are adapted to resource-poor areas. Quality care must lose its elitist status and not be viewed as a luxury good that can be afforded only by healthcare systems in resource-rich areas.
